# Left-Side Bias Is Observed in Sequential Matching Paradigm for Face Processing

**DOI:** 10.3389/fpsyg.2018.02005

**Published:** 2018-10-22

**Authors:** Chenglin Li, Qinglan Li, Jianping Wang, Xiaohua Cao

**Affiliations:** Department of Psychology, Zhejiang Normal University, Jinhua, China

**Keywords:** left-side bias, sequential matching paradigm, face perception, perceptual expertise, cognition

## Abstract

The left-side bias refers to how a chimeric face is created from the left side of a face (from the viewer’s perspective) and its mirror image are considered more similar to the original face than a chimeric face created from the right side of the same face and its mirror image. Previous studies investigated the left-side bias by using the chimeric stimuli task, where the original face and chimeric face were presented simultaneously. However, it remains unclear whether left-side bias effect is observed when the original face and chimeric face are presented sequentially. We completed two experiments using the sequential matching paradigm to investigate this issue. The results from both Experiment 1 and 2 showed that participants judged the identical proportion of the left chimeric face and original face was significantly higher than that of the right chimeric face and original face, which implies that the left-side bias effect can be observed in the sequential matching paradigm for face processing.

## Introduction

Many previous studies found that there are serial-specific expert behavioral markers for face processing including the inversion effect (e.g., Yin, 1969; Haxby et al., 1999), the composite effect (e.g., Young et al., 1987; for a review, see Richler and Gauthier, 2014), and the left-side bias effect (e.g., Gilbert and Bakan, 1973; Proietti et al., 2015). These face-selective effects indicate that the perceptual representation that we generate for faces differs from the presentation that is generated for non-face objects (for a review, see Yovel, 2016). These face-selective effects were observed in the simultaneous or sequential presentation paradigms. For example, the face inversion effect–that faces are much more difficult to recognize upside-down than other kinds of objects (Yin, 1969; for a review, see Rossion and Gauthier, 2002)–was observed when the stimuli were presented simultaneously (e.g., Taubert, 2009; de Heering et al., 2012) or sequentially (e.g., Yin, 1969; Haxby et al., 1999; for a review, see Rossion and Gauthier, 2002). Further, the composite effect, referring to the observation that recognition of the top half of a face is more difficult when the top half is aligned with the bottom half of a different face, creating the impression of a completely novel face, than when the two halves are misaligned through a lateral shift (e.g., Young et al., 1987; Hole, 1994), was observed in both simultaneous (e.g., de Heering et al., 2007; Robbins and McKone, 2007) and sequential (e.g., Haxby et al., 1999; Richler et al., 2008; for a review, see Rossion and Gauthier, 2002; Rossion, 2013) matching paradigms.

Interestingly, the left-side bias effect as another expert behavioral marker was discovered in face processing (e.g., Wolff, 1933; Brady et al., 2005; Butler and Harvey, 2005; Chung et al., 2017; Li and Cao, 2017). This posits that participantsare biased to consider that the chimeric face composed of the left side (from a viewer’s perspective) of a face is more like the original face than a chimeric face composed of the right side of the same face. Unfortunately, previous studies adopted chimeric faces that were presented simultaneously to investigate the left-side bias effect of face processing. For example, in the facial identity task, the original face and two chimeric faces (left and right) were presented simultaneously (e.g., Gilbert and Bakan, 1973; Coolican et al., 2008; Balas and Moulson, 2011; Proietti et al., 2015; Chung et al., 2017); then, participants were asked to choose which of two chimeric faces looked more like the original face, on first impression and without scrutinizing the images (e.g., Brady et al., 2005). In another frequently used task–the emotion judgment task–the two chimeric stimuli (created from a smiling half-face on one side of the vertical meridian and a neutral half-face on the other side) were also presented simultaneously (e.g., David, 1993; Failla et al., 2003; Ferber and Murray, 2005; Bourne, 2008, 2011; Innes et al., 2016). Participants made a forced-choice judgment to indicate whether the chimera with the smiling face on the left or the smiling face on the right looked happier (e.g., Coolican et al., 2008). In the above-mentioned studies, a stable left-side bias effect for face processing was observed when the original face and chimeric faces were simultaneously presented.

Importantly, the simultaneous presentation paradigm may induce diverse characteristics/functions from the sequential presentation paradigm in cognitive processes. First, the sequential presentation paradigm may increase the difficulty of the cognitive task (Ellis and Young, 1988); consequently, the performance of the same tasks differ between the sequential and simultaneous matching paradigms, such as face processing (e.g., Finley et al., 2015; Menon et al., 2015), same-different judgments (e.g., Krueger, 1983, 1984; Zhang et al., 2013), visual short-term memory (e.g., Frick, 1985; Mance et al., 2012; Becker et al., 2013; Ricker and Cowan, 2014), and judgment of geometric figures (e.g., Egeth, 1966; Nickerson, 1967; Palmer, 1978). Second, the processing strategies differ between the sequential and simultaneous presentation paradigm (e.g., Richler et al., 2009; Kolinsky et al., 2011; Wong et al., 2012; Menon et al., 2015). Especially in face processing, when the paired faces were presented sequentially, it may encourage participants to process the faces holistically. In addition, this sequential presentation paradigm reduced the amount of comparison of faces with respect to local features (e.g., Yang and Schwaninger, 2010); therefore, participants may adopt memory based-implicating strategies or the outcome-of-an-attention strategies (Richler et al., 2009, 2012; Menon et al., 2015). In contrast, in the simultaneous presentation paradigm, participants may adopt part-based, feature-matching strategies or image-matching strategies (Hole, 1994; Richler et al., 2009). Importantly, previous studies also showed that there was diverse brain function/response between the simultaneous and sequential presentation paradigm, in which humans’ right hemisphere is specialized for simultaneous, but the left hemisphere for sequential, presentation processing of information (e.g., Kimura and Durnford, 1974; Pirozzolo, 1977; Polich, 1978; Simernitskaya, 1978). Bianki (1983) found comparable results: in animals (rats), the right hemisphere is specialized for parallel (simultaneous) processing of geometrical figures, and the left hemisphere is specialized for consecutive (sequential, successive) analysis. Further, functional magnetic resonance imaging studies revealed a significant higher BOLD response to sequential than simultaneous presentation of multiple stimuli (e.g., Kastner et al., 1998; Kastner et al., 2001; Kay et al., 2013; Bernstein et al., 2014). Interestingly, regarding face processing, Shim et al. (2013) found that the BOLD responses are lower when faces are presented simultaneously than successively in ventral category-selective regions (for example, the fusiform face area and the lateral occipital complex).

Taken together, this behavior and neural evidence suggests that participants might be relying on more feature processing in the simultaneous presentation paradigm and holistic processing in the sequential presentation paradigm for face processing. Both the inversion and composite effect can be observed in simultaneous or sequential presentation paradigms, suggesting that the two effects are face-related effects, not task/strategy-related effects. In addition, previous studies showed that the left-side bias effect for face processing was only investigated with the simultaneous presentation paradigm. Evidence for the left-side bias effect in the sequential presentation paradigm was absent, implying that either the left-side bias effect for face processing is task/strategy-related, or there is a face-related effect, like the inversion or composite effect. Consequently, we adopted the classical sequential matching paradigm (e.g., Eimer et al., 2010; Fu et al., 2012; Cao et al., 2014) to clarify this issue. Based on the above-mentioned research, since the perceptual expert effects (e.g., inversion effect and composite effect) were observed in sequential matching paradigms, we expected to observe the left-side bias for face processing in the sequential matching paradigm.

## Experiment 1

### Methods

#### Participants

Participants were 40 healthy undergraduate and graduate Chinese students from Zhejiang Normal University (age range 18–28 years, mean 21.3 years, SD = 2.3; 25 females). All participants received payment for their participation and reported that they were right-handed and had normal or corrected-to-normal vision. The research protocols reported in Experiment 1 were approved by the ethical committee of Zhejiang Normal University, and written informed consent was obtained from all participants.

#### Stimuli

**FIGURE 1:**
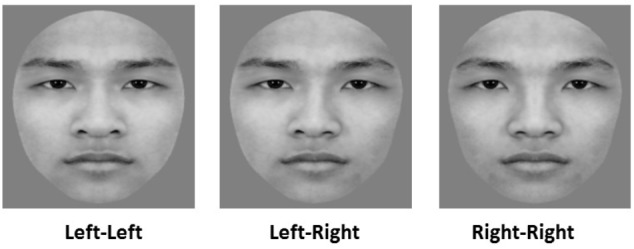
Examples of images for the chimeric face judgment task. “Left–Left” denotes a left chimeric face image, “Right–Right” denotes a right chimeric face image, and “Left-Right” denotes an original face image. This individual has given written informed consent (as outlined in Frontiers in Psychology consent form) for the publication of this image.

Sixty grayscale pictures of Chinese faces (30 female faces) were selected from a standard set of faces used in previous work by our laboratory (Cao et al., 2015). All faces were cropped into a unified oval frame to remove the external features (e.g., hair, ears, and jawline) and displayed a neutral facial expression. To investigate the left-side bias effect in face processing, we bisected each original face into two halves (left and right) along the vertical midline and combined each half-face with its mirror image to create a new chimeric face. Thus, each original face made one left and one right chimeric face. The final set of images included 60 original faces, 60 left chimeric Chinese faces, and 60 right chimeric Chinese faces (see Figure 1 for example, and refer to figure legend for consent of the participant). In the sequential matching task, there were three kinds of study stimuli–original face (O), left chimeric face (L), right chimeric face (R)–and four kinds of test stimuli: another original face that is different from the original study face (D), the same original face as the original study face (O), and a left chimeric face (L) and a right chimeric face (R) that were both from the same individual as the original study face. The study and test faces in each pair originated from the same individual except for the OD condition. To balance the trials of the “same” condition (OO, LL, RR) and those of the “different” condition (OD, OL, LO, OR, RO), there were eight matching conditions: OD, OO, LL, RR, OL, LO, OR, RO. (The former letters represent the kinds of study faces, and the latter represent the kinds of test faces.). To balance the order of original and chimeric faces, there were four matching conditions for each original face (OL, LO, OR, or RO). The numbers of trials were 60, 100, 100, 100, 60, 60, 60, and 60 for OD, OO, LL, RR, OL, LO, OR, and RO pairs, respectively. All of the stimuli subtended an angle of 4.8° × 5.1 from a viewing distance of 65 cm.

#### Procedure

The participants sat on a chair in a dimly lit room at 65 cm from the 20-inch CRT monitor (1,600 × 900-pixel resolution; 60-Hz refresh rate) on which all stimuli were presented against a light gray background. E-Prime 2.0 software was used for stimulus presentation and behavioral response collection (Psychology Software Tools, Pittsburgh, PA, United States).

**FIGURE 2:**
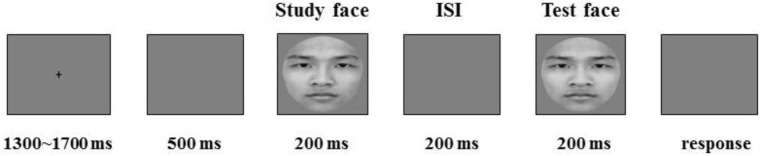
Example of the experimental procedure. The written informed consent was also obtained from the individual for the publication of this image.

The sequential matching task contained 600 trials presented randomly in eight blocks; each block consisted of 75 trials. In each trial, a fixation cross was presented for 1,300∼1,700 ms randomly in the center of the screen followed by a blank screen for 500 ms, and study and test faces were presented sequentially for 200 ms each with an intervening blank inter-stimulus interval of 200 ms. Then, the participants were asked to respond as quickly and accurately as possible by pressing the corresponding keys. The participants were told that there were three possible pair conditions: exactly the same individual paired faces, exactly different individual paired faces, and similar individual paired faces (e.g., twins’ faces). The participants were asked to press “Q” if they thought that the sequentially presented stimuli were exactly the same individual faces and “P” if they thought that the sequentially presented stimuli were different individual faces or similar twins’ faces (see Figure 2). The key assignment was counterbalanced across participants.

#### Data Analysis

After collecting data, the identical proportion and mean response time of each matching condition were collected. The identical proportion was calculated as the number of trials in which the participants judged faces to be the same divided by the total trials of each matching condition. The present study defined three matching types from same/similar matching conditions: Same type (the average of identical proportion for OO, LL, and RR); Original-Left type (the average of identical proportion for OL and LO), and Original-Right type (the average of identical proportion for OR and RO). In the sequential matching task, left-side bias refers to the significantly higher identical proportion of the Original-Left condition than that of the Original-Right condition. Moreover, both should be significantly higher than the chance level (0.5). At the same time, the response time was also analyzed for each matching type. Moreover, to compare the discriminability of the original, left chimeric, and right chimeric faces, we planned to calculate the sensitivity index (*d’*) and likelihood ratio (β) using the signal detection theory. According to the results of *d’* and β, it can further indicate whether the left-side bias effect is due to the difference in discrimination and likelihood ratio between the left and right chimeric faces. The *d’* of the original face is calculated from the hit rate (“same” responses in the OO condition) and the false alarm rate (“same” responses in OD condition). The *d’* of the left chimeric face is calculated from the hit rate (“same” responses in the LL condition) and the false alarm rate (mean of “same” responses in OL and LO conditions). The *d’* of the right chimeric face is calculated from the hit rate (“same” responses in the RR condition) and the false alarm rate (mean of “same” responses in OR and RO conditions). The βs of the original, left chimeric, and right chimeric faces are the estimated criteria of participants for the corresponding discriminability of the original, left chimeric, and right chimeric faces. All the *post hoc t*-tests statistics were computed with adjusted *p*-values.

### Results

**FIGURE 3:**
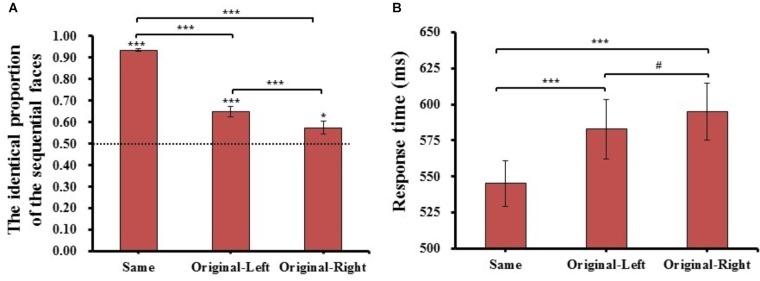
The results of identical proportion **(A)** and response time **(B)** for each matching type in Experiment 1. Error barsrepresent standard errors of the means. ^#^*p* = 0.054, ^∗^*p* < 0.05, and ^∗∗∗^*p* < 0.001.

One-way repeated-measures ANOVA of the identical proportion of the sequential faces and response time were conducted for matching type (Same, Original-Left, and Original-Right). The results are shown in Figure 3.

#### The Identical Proportion

For the identical proportion, there was a main effect of matching type (*F*_(2,78)_ = 134.870, *p* = 1.670E-21, $\eta_{P}^{2}$ = 0.78). *Post hoc t*-tests with adjusted *p*-values (*p* = 0.05/3 = 0.017, the same below) revealed that the identical proportion of Same condition (*M* = 0.93 ± 0.04) was significantly higher than that in both the Original-Left (*M* = 0.65 ± 0.16, *t*_(39)_ = 13.143, *p* = 6.575E-16, Cohen’s *d* = 1.81), and Original-Right (*M* = 0.57 ± 0.20, *t*_(39)_ = 12.833, *p* = 1.408E-15, Cohen’s *d* = 2.04) conditions. Importantly, the identical proportion in the Original-Left condition was significantly higher than that in the Original-Right condition (*t*_(39)_ = 3.987, *p* = 2.845E-04, Cohen’s *d* = 0.40), and One-sample *t*-tests comparing the proportions in both condition to the chance level (0.5) were significantly higher than the chance level (Original-Left condition: *t*_(39)_ = 5.829, *p* = 8.961E-07, Cohen’s *d* = 0.93; Original-Right condition: *t*_(39)_ = 2.381, *p* = 2.224E-02, Cohen’s *d* = 0.38). The results revealed that the participants would prefer to consider the left chimeric face more similar to the original face than the right chimeric face. This suggested that a significant left-side bias effect appeared in Chinese face processing in the sequential matching task.

#### Response Time

In analysis of the mean response time of the three matching types judged in the “same” response trials, there was a main effect of matching type (*F*_(2,78)_ = 33.615, *p =* 9.873E-12, $\eta_{P}^{2}$ = 0.46). *Post hoc t*-tests revealed the response time of the same condition (*M* = 545 ± 100 ms) was significantly faster than both Original-Left (*M* = 583 ± 124 ms, *t*_(39)_ = 6.165, *p* = 3.060E-07, Cohen’s *d* = 0.27) and Original-Right (*M* = 595 ± 130 ms, *t*_(39)_ = 6.485, *p* = 1.101E-07, Cohen’s *d* = 0.35) conditions, and the Original-Left condition was marginally faster than the Original-Right condition (*t*_(39)_ = 2.460, *p* = 1.845E-02, Cohen’s *d* = 0.09). These results showed that there was a quicker response when the participant judged the left chimeric face to be the same as the original face than the right chimeric face. The mean of identical proportions and response times for each matching condition were also reported (see Supplementary Table S1).

**FIGURE 4:**
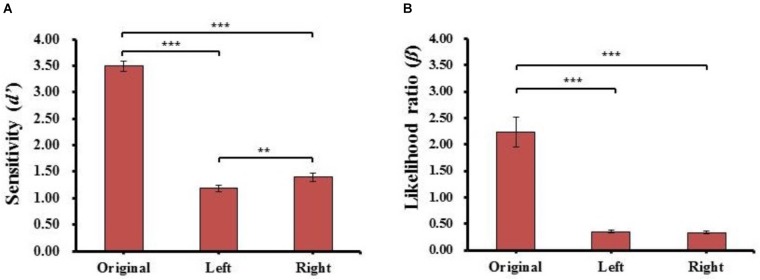
The results of sensitivity index (*d’*) **(A)** and likelihood ratio (β) **(B)** for each kind of face in Experiment 1. Error bars represent standard errors of the means. ^∗∗^*p* < 0.01, ^∗∗∗^*p* < 0.001.

One-way repeated-measures ANOVAs of the sensitivity (*d’*) and likelihood ratio (β) were conducted for face type [original face (Original), left chimeric face (Left), and right chimeric face (Right)]. The results are shown in Figure 4.

#### Sensitivity (*d’*)

For the sensitivity (*d’*), there was a main effect of face type (*F*_(2,78)_ = 405.829, *p =* 5.916E-42, $\eta_{P}^{2}$ = 0.91), *post hoc t*-tests revealed the sensitivity (*d’*) of the original face (*d’*_Original_ = 3.49 ± 0.64) was significantly higher than the sensitivity (*d’*) of both left (*d’*_Left_ = 1.18 ± 0.43, *t*_(39)_ = 24.951, *p* = 1.383E-25, Cohen’s *d* = 4.14) and right (*d’*_Right_ = 1.40 ± 0.47, *t*_(39)_ = 21.389, *p* = 3.769E-23, Cohen’s *d* = 3.67) chimeric faces. Moreover, the sensitivity (*d’*) of the right chimeric face was significantly higher than the sensitivity (*d’*) of the left chimeric face (*t*_(39)_ = 2.744, *p* = 9.135E-03, Cohen’s *d* = 0.47). The results reveal that the strongest discriminability occurred with the different original face, especially, the discriminability was stronger between the right chimeric and original faces than between the left chimeric and original faces. The results further showed the participants considered the left chimeric face more similar to the original face than the right chimeric face.

#### Likelihood Ratio (β)

For likelihood ratio (β), there was a main effect of face type (*F*_(2,78)_ = 46.819, *p* = 4.382E-14, $\eta_{P}^{2}$ = 0.57), *post hoc t*-tests revealed the estimated criteria (β) of the original face (β_Original_ = 2.23 ± 1.79) were significantly higher than the estimated criteria (β) of both the left (β_Left_ = 0.35 ± 0.19, *t*_(39)_ = 6.758, *p* = 4.607E-08, Cohen’s *d* = 1.34) and right chimeric faces (β_Right_ = 0.34 ± 0.17, *t*_(39)_ = 6.958, *p* = 2.444E-08, Cohen’s *d* = 1.20), but there was no significant difference between the estimated criteria (β) of the left and right chimeric faces (*t*_(39)_ = 0.721, n.s.). These results suggested that the participants had different discriminability between left and right chimeric faces with the same estimated criteria. To carry more information of each participant, the individual results of identical proportion and response time for each matching type, and sensitivity index (d′) and likelihood ratio (β) for each kind of face in Experiment 1 also plotted (see Supplementary Figure 1).

## Experiment 2

Experiment 1 demonstrated that there is a left-side bias effect for face processing in the sequential matching paradigm. However, in Experiment 1, the study and test stimuli were presented at the same location, and the participants may have been affected by perceptual afterimages or adopted a screen position-based strategy. Therefore, in Experiment 2, the test face was randomly moved 20 pixels to the left or right of the center to minimize afterimages and to prevent a strategic, screen position-based approach to the sequential matching tasks (Collova et al., 2017).

### Methods

#### Participants

Forty healthy Chinese students (age range 18–27 years, mean 20.8 years, SD = 2.4; 27 females) participated in Experiment 2. The selection criteria were the same as in Experiment 1. None had participated in Experiment 1. The research protocols reported in Experiment 2 were approved by the ethical committee of Zhejiang Normal University, and written informed consent was obtained from all participants.

#### Stimuli

The stimuli used in Experiment 2 were the same as in Experiment 1.

#### Procedure

The laboratory setup and task were the same as those in Experiment 1 except the test stimuli were randomly moved 20 pixels to the left or right of the center.

### Results

#### The Identical Proportion

**FIGURE 5:**
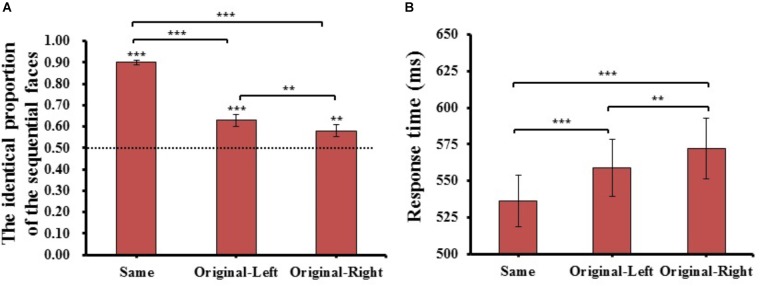
The results of identical proportion **(A)** and response time **(B)** for each matching type in Experiment 2. Error bars represent standard errors of the means. ^∗∗^*p* < 0.01, ^∗∗∗^*p* < 0.001.

The method of data analysis was the same as in Experiment 1. One-way repeated-measures ANOVA of the identical proportion of the sequential faces and response time were conducted for matching type (Same type, Original-Left type, and Original-Right type). The results are shown in Figure 5. For the identical proportion of the sequential faces, there was a main effect of matching type (*F*_(2,78)_ = 146.086, *p* = 4.207E-27, $\eta_{P}^{2}$ = 0.79), *post hoc t*-tests revealed that the identical proportion of the Same condition (*M* = 0.90 ± 0.07) was significantly higher than both Original-Left (*M* = 0.63 ± 0.18, *t*_(39)_ = 12.014, *p* = 1.108E-14, Cohen’s *d* = 1.41) and Original-Right conditions (*M* = 0.58 ± 0.18, *t*_(39)_ = 14.893, *p* = 1.108E-17, Cohen’s *d* = 1.65); importantly, the Original-Left condition was significantly higher than the Original-Right condition (*t*_(39)_ = 3.148, *p* = 3.144E-03, Cohen’s *d* = 0.27); and both the similar matching conditions (Original-Left, Original-Right) were significantly higher than chance level (0.5) (Original-Left condition: *t*_(39)_ = 4.621, *p* = 4.103E-05, Cohen’s *d* = 0.74; Original-Right condition: *t*_(39)_ = 2.985, *p* = 4.871E-03, Cohen’s *d* = 0.48). The results were consistent with the results of Experiment 1, showing that participants tend to consider the left chimeric face more similar to the original face than the right chimeric face.

#### Response Time

For the mean response time of the three matching types judged as the same, there was a main effect of matching type (*F*_(2,78)_ = 22.703, *p* = 1.696E-08, $\eta_{P}^{2}$ = 0.37), *post hoc t*-tests revealed the Same condition (*M* = 536 ± 112 ms) was significantly faster than both Original-Left (*M* = 559 ± 123 ms, *t*_(39)_ = 4.497, *p* = 6.040E-05, Cohen’s *d* = 0.19) and Original-Right conditions (*M* = 572 ± 132 ms, *t*_(39)_ = 5.615, *p* = 1.771E-06, Cohen’s *d* = 0.26), and the Original-Left condition was significantly faster than the Original-Right condition (*t*_(39)_ = 2.839, *p* = 7.153E-03, Cohen’s *d* = 0.10). The results showed that there was a quicker response when participants judged the left chimeric face as more similar to the original face than the right chimeric face. The mean of identical proportions and response times for each matching condition were also reported (see Supplementary Table S1).

#### Sensitivity (*d’*)

**FIGURE 6:**
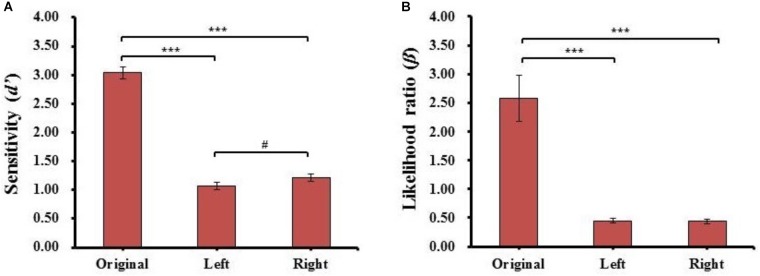
The results of sensitivity index (*d’*) **(A)** and likelihood ratio (β) **(B)** for each kind of face in Experiment 2. Error bars represent standard errors of the means. #*p* = 0.063, ^∗∗∗^*p* < 0.001.

One-way repeated-measures ANOVA of the sensitivity (*d’*) and likelihood ratio (β) conducted for face type was the same as in Experiment 1. The results are shown in Figure 6. For the sensitivity (*d’*), there was a main effect of face type (*F*_(2,78)_ = 264.717, *p* = 1.719E-35, $\eta_{P}^{2}$ = 0.87), *post hoc t*-tests revealed the sensitivity (*d’*) of the original face (*d’*_Original_ = 3.04 ± 0.68) was significantly higher than the sensitivity (*d’*) of both the left (*d’*_Left_ = 1.07 ± 0.41, *t*_(39)_ = 18.533, *p* = 6.178E-21, Cohen’s *d* = 3.40) and right chimeric faces (*d’*_Right_ = 1.21 ± 0.40, *t*_(39)_ = 16.307, *p* = 5.197E-19, Cohen’s *d* = 3.20), and the sensitivity (*d’*) of the right chimeric face was marginally higher than the sensitivity (*d’*) of the left chimeric face (*t*_(39)_ = 2.401, *p* = 2.120E-02, Cohen’s *d* = 0.35). The results similar with those of Experiment 1; the discriminability was stronger between the right chimeric and original faces than between the left chimeric and original faces.

#### Likelihood Ratio (β)

For likelihood ratio (β), there was a main effect of face type (*F*_(2,78)_ = 30.463, *p* = 1.671E-10, $\eta_{P}^{2}$ = 0.44), and *post hoc t*-tests revealed that the estimated criteria (β) of the original face (β_Original_ = 2.57 ± 2.52) were significantly higher than the estimated criteria (β) of both left (β_Left_ = 0.45 ± 0.24, *t*_(39)_ = 5.505, *p* = 2.520E-06, Cohen’s *d* = 0.97) and right chimeric faces (β_Right_ = 0.43 ± 0.24, *t*_(39)_ = 5.543, *p* = 2.231E-06, Cohen’s *d* = 0.97), but there was no significant difference between the estimated criteria (β) of the left and right chimeric faces (*t*_(39)_ = 0.734, n.s.). Combined with the results of discriminability, this suggests that the participants had different discriminability between left and right chimeric faces with the same estimated criteria. For the individual results of Experiment 2, see Supplementary Figure 2.

## Discussion

We investigated whether there is a left-side bias in face processing by using a sequential matching paradigm. Consistent with previous studies using simultaneous presentation (e.g., Luh et al., 1991; Burt and Perrett, 1997; Coolican et al., 2008; Proietti et al., 2015; Chung et al., 2017), our results showed that participants judged the identical proportion of the left chimeric face and original face as significantly higher than that of the right chimeric face and original face, which suggests that a reliable left-side bias effect for face processing was observed in the sequential matching paradigm.

Interestingly, the inversion effect and composite effect for face processing were observed in both simultaneous and sequential matching tasks (e.g., Yin, 1969; Haxby et al., 1999; de Heering et al., 2007, 2012; Robbins and McKone, 2007; Richler et al., 2008; Taubert, 2009; for a review, see Rossion and Gauthier, 2002; Rossion, 2013). Importantly, the left-side bias for face processing was observed in previous studies by using simultaneous matching tasks (e.g., Brady et al., 2005; Coolican et al., 2008; Li and Cao, 2017) and by using sequential matching tasks in the present study. The prior results combined with our results indicate that three expert behavioral markers (e.g., the inversion effect, composite effect, and left-side bias effect) for face processing are observed stably when faces are presented simultaneously or sequentially, which suggests that all the expert behavioral markers for face processing are face-related, not task/strategy-related processing.

In both Experiments 1 and 2, the results of the identical proportion of the paired faces showed that participants considered the left chimeric face as more like the original face than the right chimeric face. Importantly, these results demonstrated that the sequential matching paradigm is an appropriate paradigm to test the left-side bias effect, and extended the effect in the sequential presentation. This paradigm creates an opportunity to compare, systematically, distinct presentation sequences (sequential or simultaneous matching) to elucidate the left-side bias of face perception. Moreover, our results of response time showed that there was a response time bias for the left chimeric face. For the “same” response trials, participants responded to the Original-Left condition more quickly than to the Original-Right condition. This was consistent with previous research (e.g., Bourne, 2008; Butler and Harvey, 2008). Interestingly, the results showed that participants had different discriminability for the left and right chimeric face processing, but with the same estimated criteria. Participants had lower discriminability between the original face and the left chimeric face than between the original face and the right chimeric face in same estimated criteria, which may explain the cause of the left-side bias in face processing. Namely, the low discriminability should induce more similarity judgment between the left chimeric face and the original face.

The previous results regarding simultaneous matching tasks and our results show that the left-side bias effect for face processing is stable when faces are presented simultaneously/sequentially. Previous studies suggested that participants may employ diverse strategies in the simultaneous presentation face processing from that of the sequential matching task (Richler et al., 2009; Menon et al., 2015). For example, participants can repeatedly scan images when both faces are presented simultaneously, and this presentation sequence encourages a feature-matching strategy or image-matching strategy (Hole, 1994; Richler et al., 2009; Menon et al., 2015). However, when the faces are presented sequentially, participants must represent the presented faces in memory (Richler et al., 2009; Richler et al., 2012; Menon et al., 2015). This presentation sequence may encourage a memory-based implicating strategy. Therefore, it suggests that both the distinct processing strategies used in the simultaneous or sequential matching task can obtain the stable left-side bias effect, which implies that the left-side bias effect is not strategy-related face processing.

More importantly, previous studies also showed that there are diverse brain functions between sequential and simultaneous presentation, in which humans’ right hemisphere is specialized for simultaneous, but the left hemisphere for sequential, presentation processing of information (e.g., Kimura and Durnford, 1974; Pirozzolo, 1977; Polich, 1978; Simernitskaya, 1978). The previous studies suggested that the left-side bias effect may be an indicator of right hemisphere dominance in face processing (e.g., Megreya and Havard, 2011). Importantly, several recent studies have suggested important functional roles of the left hemisphere in face perceptual learning/processing (e.g., Meng et al., 2012; Bi et al., 2014; Goold and Meng, 2017). Therefore, future studies should investigate the relationship of the left hemisphere with the facial left-side bias effect. The prior results combined with our results indicate that this effect can be observed stably when chimeric faces are presented simultaneously or sequentially. It suggests that the left-side bias may be a face-related effect, not a task/strategy-related effect, like the inversion or composite effect. Interestingly, the present study also demonstrated that the sequential presentation is an impactful paradigm to investigate the left-side bias effect. Because the simultaneous presentation paradigm is not an appropriate paradigm to investigate the brain mechanism underlying the left-side bias effect in face processing, the sequential presentation paradigm used in our study provided an appropriate paradigm that directly investigated the brain mechanism for the left-side bias effect in face processing.

The current study had some limitations. For example, we did not use eye-tracking techniques. Previous eye-tracking studies showed that the first saccades after image presentation are biased to the left-side for all kind of images (e.g., for a review, see Ossandón et al., 2014). Further, a similar pattern was found in chimeric (half female, half male) face processing: a greater number of left fixations than that of right fixations (e.g., Butler et al., 2005; Butler and Harvey, 2006). This would be especially important for a sequential matching task like the one used in the present study, where there is plenty of time for participants to make a left-side saccade. Therefore, the left-side bias effect observed in sequential matching task might potentially result in participants comparing only face parts that are in the left hemifield. Therefore, future studies should investigate this issue by employing the eye-tracking technique.

In addition, our studies just tested a kind of exposure duration (e.g., 200 ms) of face stimuli. Previous studies showed that the left-side bias was affected by exposure duration of face stimuli in simultaneous presentation paradigms (e.g., David, 1993; Butler and Harvey, 2006, 2008). In the sequential presentation task for face processing, the exposure duration of face stimulus was also a crucial factor that affected face perception (e.g., Zago et al., 2005; Feuerriegel et al., 2015). Therefore, future studies should systematically examine how those factors (e.g., exposure duration, inter-stimulus interval, etc.) affect the degree of left-side bias for face processing in the sequential presentation paradigm.

## Author Contributions

CL and XC designed the experiments, performed the data analysis, and wrote the manuscript. CL, QL, and JW executed the project.

## Conflict of Interest Statement

The authors declare that the research was conducted in the absence of any commercial or financial relationships that could be construed as a potential conflict of interest.
