# Supplementary material for: Left-Side Bias Is Observed in Sequential Matching Paradigm for Face Processing
Source: Front Psychol. 2018 Oct 22;9:2005. doi: 10.3389/fpsyg.2018.02005 (PMC6204458; doi:10.3389/fpsyg.2018.02005)

**Figure and Table Captions**

Table 1. The mean of identical proportions and response times for each matching condition of Experiment 1 and 2. “Exp 1” denotes Experiment 1. “Exp 2” denotes Experiment 2. “OO, LL, RR, OL, LO, OR, RO” denote the corresponding matching condition conditions.

Figure 1. The individual results of identical proportion (A) and response time (B) for each matching type, and sensitivity index (*d’* ) (C) and likelihood ratio (*β*) (D) for each kind of face in Experiment 1.
Figure 2. The individual results of identical proportion (A) and response time (B) for each matching type, and sensitivity index (*d’* ) (C) and likelihood ratio (*β*) (D) for each kind of face in Experiment 2.

Table 1 The mean of identical proportions and response times for each matching condition(M±SD).

|  |  | OO | LL | RR | OL | LO | OR | RO |
| --- | --- | --- | --- | --- | --- | --- | --- | --- |
| Exp 1 | Identical proportion | 0.93±0.05 | 0.93±0.05 | 0.94±0.04 | 0.65±0.16 | 0.65±0.17 | 0.57±0.20 | 0.58±0.20 |
|  | Response time (ms) | 552±106 | 547±98 | 536±100 | 582±121 | 584±128 | 592±131 | 598±131 |
| Exp 2 | Identical proportion | 0.89±0.09 | 0.90±0.08 | 0.91±0.07 | 0.63±0.18 | 0.64±0.19 | 0.58±0.18 | 0.59±0.19 |
|  | Response time (ms) | 546±116 | 534±113 | 527±109 | 560±129 | 558±123 | 583±141 | 561±127 |

**Figure 1**


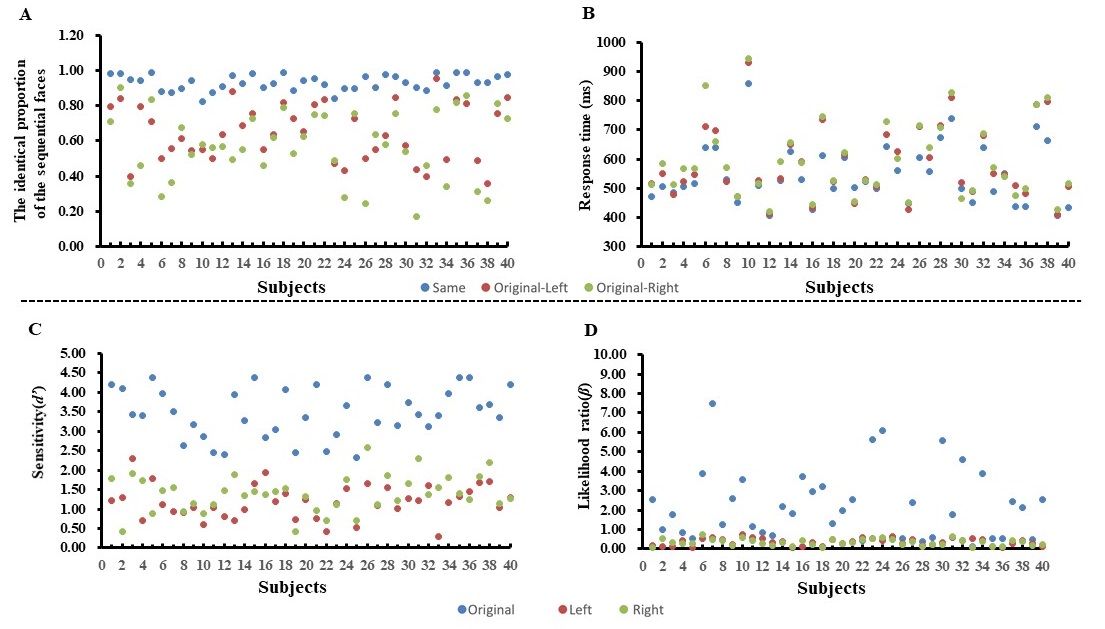


**Figure 2**
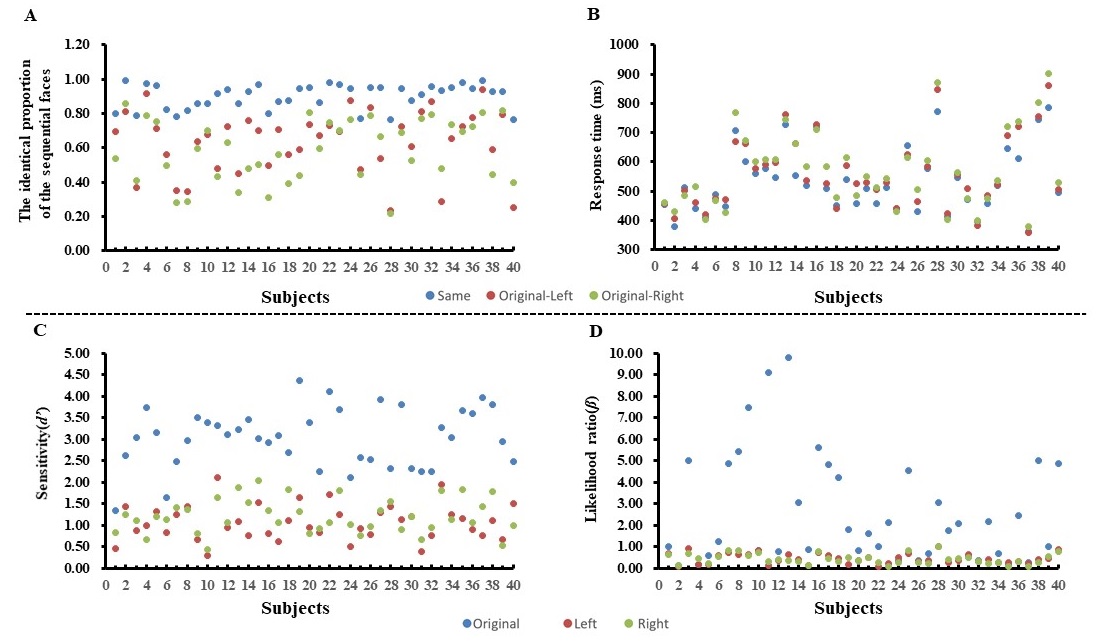

Supplement: Supplementary file 1 [file Table_1.DOCX]
